# Beliefs and Attitudes of British Residents about the Welfare of Fur-Farmed Species and the Import and Sale of Fur Products in the UK

**DOI:** 10.3390/ani12050538

**Published:** 2022-02-22

**Authors:** Carly Halliday, Steven P. McCulloch

**Affiliations:** Centre for Animal Welfare, University of Winchester, Winchester SO22 4NR, UK; C.Halliday1.16@unimail.winchester.ac.uk

**Keywords:** animal welfare, Brexit, COVID-19, fur trade, fur farming, prohibition, public attitudes

## Abstract

**Simple Summary:**

Fur farming has become increasingly controversial in the western world. Fur-farmed species such as mink contracting and transmitting COVID-19 have put a further spotlight on the industry. The United Kingdom (UK) and other nations have banned fur farming due to welfare concerns. Despite this, due to European Union (EU) membership, the UK has continued to import and sell fur from two million animals each year. The UK left the EU in 2020 and the British government is now considering a ban on the import and sale of fur. This paper reviews public polls on British attitudes to the fur industry conducted between 1997 and 2021. The polls reveal consistently high majority opposition to the fur industry. The paper then reports the results of a new questionnaire to explore in further depth the views of UK residents on the fur industry and attitudes to a ban. A large majority (86%) believed fur-farmed animals do not experience a good life. A similar majority (83%) believed it was unacceptable for the UK government to ban fur farming and continue to import and sell fur from producers overseas. Over three quarters (78%) supported a legal ban on the import and sale of fur in the UK.

**Abstract:**

Around 100 million animals are killed annually for the global fur trade, with 85% reared on fur farms and 15% trapped in the wild. Fur farming is banned across the United Kingdom (UK) under the Fur Farming (Prohibition) Act 2000 in England and Wales and parallel legislation in Scotland and Northern Ireland. Despite the farming bans, the import and sale of fur products to the UK have continued, largely due to European Union (EU) membership. The UK left the EU in 2020 and the British government is exploring a potential ban on the import and sale of fur post-Brexit. This paper reviews public surveys on attitudes to fur farming in the UK from 1997 to 2021. It then reports the results of an online questionnaire to investigate in greater depth the beliefs of UK residents (*n* = 326) about the welfare of animals used in fur production, knowledge of the legal context of the fur trade and attitudes toward a ban on the import and sale of fur in the UK. A large majority (86%) of respondents believed that fur-farmed animals do not experience a good life. Over four-fifths (83%) disagreed that it is morally acceptable for the UK government to ban fur farming and yet continue to import and sell fur from producers overseas, with over three-quarters (78%) supporting a legal ban on the import and sale of fur in the UK.

## 1. Introduction

COVID-19 has caused devastating impacts on public health and human society since cases of a new virus were first identified in China in 2020 [1]. SARS-CoV-2, the virus that causes COVID-19, is believed to have originated in bats and transmitted to humans through an intermediary species in a wet market in Wuhan, China [2]. COVID-19 is, therefore, a zoonotic disease, meaning it can be transmitted from vertebrate animals to humans. Mustelids, including mink (*Neovison vison*), are particularly susceptible to SARs-CoV-2 [3]. Since 2020, there have been outbreaks on 400 mink farms in Europe and America. Raccoon dogs (*Nyctereutes procyonoides*) and red foxes (*Vulpes vulpes*) are also susceptible to coronaviruses.

A risk assessment conducted by the global tripartite of the United Nations Food and Agriculture Organisation of the United Nations (UN FAO), the World Organisation for Animal Health (OIE) and the World Health Organisation (WHO) revealed a high risk of introducing SARS-CoV-2 from fur farms to humans and wildlife populations [4]. There have been over 200 human COVID-19 cases in humans linked to fur farms. The outbreaks on European fur farms have led to the culling of over 20 million animals. Animal protection organisations have argued that the poor conditions on fur farms make them ideal breeding grounds for COVID-19, and called for a global end to fur farming ahead of global summits such as the G7 meeting [5].

The threat to human health posed by fur farms has led the Dutch Government to bring its ban on fur farming forward from 2024 to 2020 [6]. France, Italy and Ireland are also legislating against fur farming in part due to COVID-19. These COVID-19-related bans follow legal prohibitions on fur farming for welfare reasons in many other European states. These include the UK (2002), Austria (2005) and Croatia (2018). Germany (2017), Sweden (2005, 2014) and Switzerland (2008), meanwhile, have introduced stricter welfare regulations. In some cases, for example, in Germany, this has led to the closure of fur farms as they are no longer profitable [7].

Around 85% of fur sold globally comes from animals that are raised on farms and around 15% comes from trapping or hunting wild animals [8]. Species commonly farmed for their fur include mink (*Neovison vison*), the red fox (*Vulpes vulpes*), arctic fox (*Vulpes lagopus*), rabbit (*Oryctolagus cuniculus*), raccoon dog (*Nyctereutes procyonoides*) and chinchilla (*Chinchilla lanigera*). North America and Canada are the largest producers of wild furs and muskrat (*Ondatra zibethicus*), raccoon (*Procyon lotor*), sable (*Martes zibellina*), beaver (*Castor*), coyote (*Canis latrans*), bobcat (*Lynx rufus*) and lynx (*Lynx*) are some of the most common species that are trapped or hunted for their fur [9].

The Fur Information Council of America has estimated that the world’s total fur retail sales in 2019 were €22 billion. According to the Chinese Leather Industry Association, China is the world’s largest producer and consumer of fur, as well as the world’s largest fur-processing and trading country, producing a total of 20.73 million mink, 17.39 million fox and 12.33 million raccoon dog skins in 2018 [10]. Around 50% of global fur production comes from Europe, with 5000 farms operating across 22 European countries [11]. In the US, wild fur represents a significant part of total fur production, and in 2015, over 3.5 million wild fur skins were produced with a production value of 53 million USD [12]. 

The fur industry has come under widespread criticism in the UK and across the Western world since the second half of the 20th century [13,14,15]. Those who oppose it argue that the fur farming model of intensive confinement battery cage systems is inherently inhumane and that the fur industry’s attempts to create welfare certification schemes (e.g., Origin Assured, WelFur, FurMark) provide only superficial adjustments to a system that is fundamentally unable to provide animals with a life worth living [16]. Cage sizes vary slightly by country, but according to WelFur, they are typically 90 cm deep × 30 cm wide for mink and 0.8–1.2 m^2^ for foxes. A report by the EU Scientific Committee on Animal Health and Welfare (SCAHAW) [17] into the welfare of animals kept for fur production concluded that typical mink and fox cages impair animals’ welfare because they do not meet their important needs.

The methods used to restrain and kill wild animals for their fur have significant welfare implications and the ability to cause immense suffering and pain. The leg-hold trap can cause substantial suffering, the duration and severity of which depend on the time taken to retrieve the animal from the trap and the manner of its death [18]. Wild animals may remain trapped for extended periods before being found and killed, without access to food or water, and experience both psychological and physiological stress [18]. Through the Pests Act (1954), the UK banned the use of the leg-hold trap, and in 1991, Council Regulation (EEC) no. 3254/91 prohibited the use of leg-hold traps and imports of fur products from animals caught using leg-hold traps. This prompted threats of trade disputes from fur-exporting nations, and as a result, the International Agreement on Humane Trapping Standards was agreed on between the EU, Canada and the Russian Federation in 1998, taking effect in 2008. It provided, amongst other things, for the continued trade of fur products caught using ‘approved humane’ trapping methods, including the padded leghold trap, and drowning traps for beavers. The humaneness of such traps has been questioned [19].

The UK government banned fur farming in the early 21st century following anti-fur campaigns in the 1980s and 1990s. The decision for a ban was informed in part by a report from the UK’s Farm Animal Welfare Council (FAWC), which concluded that fur farms could not satisfy some of the most basic needs of the animals kept in them, in particular, their freedom to display normal patterns of behaviour [20]. In 2000, fur farming was banned in England and Wales under the Fur Farming (Prohibition) Act. The devolved administrations in Scotland and Northern Ireland followed with parallel legislation, which ensured fur farming was prohibited across the UK. Scotland passed the Fur Farming (Prohibition) Act in 2002, and Northern Ireland the Fur Farming (Prohibition) Order in 2002.

Despite the bans on fur farming, fur products continue to be imported and sold in the UK. Humane Society International UK (HSI UK) [16] has estimated that the UK imports the equivalent of two million whole furs each year. The import and sale of fur continue partly because the UK has been a member of the European Union (EU). McCulloch [21] has summarised the political context of the UK fur industry in a paper discussing the opportunities Brexit presents for animal welfare. In the EU, there is free movement of goods, capital, services and labour in the single market. Animals and their products are legally considered as property and traded dead or alive as commodities. It is likely the UK would have been challenged at the European Court of Justice by fur-producing member states had it implemented a unilateral ban as a member of the EU. The UK formally left the EU in January 2020, and Brexit, therefore, represents an opportunity for the UK to ban the import and sale of fur products in the UK [21].

The UK Parliamentary Environment, Food and Rural Affairs (EFRA) Committee published an inquiry into the fur trade in 2018 [22]. The Parliamentary Committee ultimately recommended that the Government hold a public consultation on whether to ban the import and sale of fur. In its response to the petition, the Government stated the following:

*There will be an opportunity for government in the future, once we have left the EU and the nature of our future trading relationship has been established, to consider further steps such as a ban on fur imports or a ban on sales* [23].

Later in 2018, a UK Government and Parliament petition was submitted to ‘Ban the sale of animal fur in the UK’ [24]. The petition refers to São Paulo banning the import and sale of fur in 2015 and India banning the import of mink, fox and chinchilla fur in 2017. In 2021, Israel became the first country to ban the sale of fur. In the US, California banned the sale of fur in 2019. This followed bans on the sale on the sale of fur in the US cities of Los Angeles, San Francisco, Berkeley and West Hollywood [7].

The 2018 UK petition received 109,553 signatures from the British public. Since the petition received over 100,000 signatures, it was debated in Westminster Hall of the UK Parliament in June 2018. The Government responded to the petition in November 2018. It stated that it would work at an international level to agree on global animal welfare standards to phase out inhumane farming and trapping methods [24].

In its 2021 Action Plan for Animal Welfare, the UK Government acknowledged that fur farming is banned in the UK. It then stated that it will explore ‘potential action’ in this area [25]. Later in 2021, the UK Government launched a consultation on the Fur Market in Great Britain [26]. The consultation requested views on respondents’ attitudes to the import and sale of fur products in Great Britain. At the time of writing in January 2022, the Government is yet to publish a summary of the consultation response.

This research investigates the beliefs and attitudes of UK residents toward the fur trade. The research questions are as follows:

1. What are UK residents’ beliefs about the welfare of fur-farmed and wild-trapped animals?
2. What are UK residents’ beliefs about the legal context of the fur trade in the UK?
3. What are UK residents’ attitudes toward the import and sale of fur in the UK, and do they support regulation or prohibition?

The research employed a quantitative-based questionnaire to investigate the research questions, and this paper reports the results of the questionnaire.

Section 2 of this paper reviews surveys on attitudes to the fur industry in the UK from 1997 to 2021. Section 3 describes the questionnaire-based methodology for the research that forms the main part of this paper. The questionnaire investigated the beliefs of UK residents about the welfare of animals killed in the wild and farmed for their fur, their awareness of the current UK ban on fur production, and their attitudes toward a potential ban on the import and sale of fur products in the UK. The results of the questionnaire are presented and discussed in Section 4 and Section 5, respectively.

## 2. Public Attitudes to Fur in the UK

Opposition to fur farming and the use of animals for fur has been consistently high in the UK since the late 20th century. Table 1 summarises major surveys conducted in the UK between 1997 and 2021.

The findings of the surveys summarised in Table 1 reveal that in the UK, public opposition toward the use of fur has been consistently high since 1997, and that wearing fur products is unacceptable to a substantial majority of people. A high proportion of those surveyed between 1997 and 2021 stated they would refuse to wear real fur. An Ipsos MORI poll [27] revealed that 87% of adults would never wear real fur, and of those, 73% thought it wrong or disapproved of wearing fur. In a 2014 YouGov poll, 74% of participants also believe the use of animals for the production of fur for the fashion industry was wrong [30]. A 2010 survey found that 95% of people would refuse to wear real fur [29]. The most recent YouGov poll surveyed found that 93% of people did not wear fur [33]. In the 2016 YouGov poll, rabbit fur received the highest approval rating, although it was still only acceptable to one in five people [31]. In the 2007 RSPCA poll, 61% of participants believed there was a moral difference between animals farmed for meat and animals raised for fur [28]. This raises questions about the public attitude toward using animals for their fur when perhaps considered a by-product of the meat industry, along with their beliefs and understanding of the welfare of animals kept in farmed environments for food production.

As revealed in the 2016 YouGov poll, less than 10% of respondents found it acceptable to buy or sell products containing domestic dog, cat or seal fur. Dogs and cats are commonly kept as companion animals; we generally have a stronger relationship with these species and laws protect them to a greater extent. Furthermore, seal pups are widely considered aesthetically pleasing. Their method of slaughter by being clubbed to death has been well-publicised and is considered by many to be brutal. For these reasons, the 2016 YouGov poll results on cat, dog and seal fur were perhaps not surprising. The acceptability of using wild animals for their fur was also low: only 8–12% of respondents found it acceptable to buy or sell fur from foxes, mink, chinchilla, raccoon dogs and coyotes [31].

The 2018 YouGov poll found that over two-thirds of respondents (69%) would support a ban on the import and sale of fur in the UK [32]. The 2020 YouGov poll then revealed a percentage in support of a ban that was even slightly higher (72%), with only 16% of people opposed. The results of the surveys also show potential support for the introduction of labelling schemes for fur, with a large proportion of participants believing that fur products should be labelled as real or fake. The results of both the 2007 RSPCA and 2011 TNS poll showed that over 90% of respondents supported stronger labelling of fur products [28,29]. 

At the time of writing in January 2022, one of the most recent polls (*n* = 2026) reveals that 72% of the British public support a ban on the import and sale of animal fur in the UK, while 12% are opposed and 3% do not know [34]. A further poll (*n* = 1647) from 2021 reveals that 93% of respondents considered it unacceptable to keep foxes for their whole lives in wire cages measuring 1–1.5 m^2^, 92% considered it unacceptable to trap wild animals (e.g., coyotes) in leg-hold traps and 92% also considered it unacceptable to kill foxes by anal/vaginal electrocution [35].

## 3. Methodology

### 3.1. Questionnaire

A questionnaire comprising 36 closed questions and one open question was used in this research (see Supplementary File S1). The questionnaire consisted of three sections: section 1 collected demographic data, section 2 was concerned with beliefs about the welfare of animals killed for their fur and section 3 was about attitudes toward fur and the import and sale of fur in the UK. Sections 2 and 3 used Likert scales and the points on each Likert scale were labelled with a description of what they represented (e.g., ‘strongly agree’, ‘strongly disagree’). The survey items from sections 2 and 3 are listed in Table 2.

The questionnaire was published using SurveyMonkey and could be filled out from August to September 2018. It was first piloted on colleagues in animal welfare before launching. Feedback was incorporated into the questionnaire to improve it before publishing it online. A link to the questionnaire was generated and distributed on the social media platforms Facebook and Twitter. Two inclusion criteria had to be met to take part in the survey. Firstly, respondents needed to be residents of the UK, and secondly, respondents had to be aged 18 years or over. Ethical approval for conducting the research was received from the University of Winchester in the UK.

### 3.2. Data Cleaning and Analysis

Once participants had completed the survey, the results were collated and exported from SurveyMonkey into Microsoft Excel. Responses were cleaned to remove incomplete entries and those that did not meet the inclusion criteria. Responses were excluded if respondents skipped multiple sections or did not provide sufficient demographic data. Respondents who stated that they did not reside in the UK were also excluded. IBM SPSS Statistics 25 and 26 were used to analyse the data. Chi-squared goodness-of-fit tests were performed on the data derived from sections 2 and 3 to assess whether the differences in responses were statistically significant.

After data cleaning, there were a total of 326 responses included in the results. Of the study sample, 84% were female and 16% were male. The ages of respondents ranged from 20 to 80+ years, though the most common age bracket was 30–39 years at 37.5%. Most respondents lived in the Southeast region (38.7%, 127). The majority of respondents had either a bachelor’s (44%) or master’s degree (17.1%).

### 3.3. Limitations of the Study

The major limitations of the study relate to the sample size and the self-selected nature of the sample. After data cleaning, the sample totalled 326 respondents. This compares to sample sizes of around 1500–2000 in the UK polls on British attitudes to fur farming summarised in Table 1 of this paper. Furthermore, the polls in Table 1 were generally conducted by large polling companies using representative samples in terms of demographics. In contrast, the questionnaire reported in this paper was based on a self-selected sample that was, therefore, non-representative. Consistent with most self-selected surveys, female respondents were overrepresented in this study, with 84% of respondents being female and 16% male. The overrepresentation of female respondents in the study risks biasing the survey, particularly because research in animal welfare often reports that females have greater concerns and more progressive views on welfare [36,37].

Despite the above-mentioned clear limitations of the survey, the strength of the survey relates to exploring timely, important issues related to attitudes to fur farming in greater depth. The polls reviewed in the earlier part of this paper are representative of the general population. Despite this, they generally only ask a few simple questions so are limited in depth. In contrast, this survey provides insight by investigating a broader and deeper range of issues. These include respondents’ beliefs about the welfare of fur-farmed and wild-trapped animals, beliefs about the legal context of the fur trade in the UK and attitudes to the import and sale of fur in the UK.

In many cases, the reliability of the results reported in this paper is boosted when they are considered alongside those of the surveys from 1997–2000 that are summarised in Table 1. For instance, the 2018 HSI UK-commissioned poll conducted by YouGov found that 69% of the British public would support a ban on the import and sale of all animal fur in the UK [32]. Then, an HSI UK-commissioned YouGov poll from 2020 found that 72% of the British public would support a ban [33]. Similarly, the survey in this study found that 78% supported a legal ban on the import and sale of fur in the UK. Hence, the findings from this study in many cases corroborate other surveys, which improves the reliability of our findings. At the same time, the slightly higher figure of 78%, compared to 69% (2018) and 72% (2020), probably relates to the unrepresentative sample with a higher proportion, in particular, of female respondents, who as we mentioned, have more progressive views about animal welfare.

## 4. Results

### 4.1. Beliefs of UK Residents about the Welfare of Fur-Farmed and Wild-Trapped Animals

Table 3 illustrates respondents’ beliefs about the welfare of fur-farmed and wild-trapped animals. Nearly three-quarters (72.3%) disagree that animals on fur farms have a life worth living. A much smaller proportion (4.3%) believe they have a good life. Nearly four-fifths (77.8%) believe animals on fur farms are not killed humanely, with 12.7% of respondents unsure and 9.5% believing they are killed humanely. Three-quarters (75.2%) of respondents do not believe that welfare standards on fur farms are well-regulated, with only 6.2% believing they are well-regulated and 18.6% remaining neutral. Three-quarters (75.2%) of respondents believe that fur farms cannot meet the welfare needs of species such as mink and foxes. Nine out of ten (90.9%) believe that cages cannot provide species such as mink and foxes with a good level of welfare. In terms of wild animals, almost all respondents (96.4%) believe leg-hold traps cause suffering to species such as the coyote and lynx. Only 3.6% believe animals caught in traps are killed humanely. Chi-squared goodness-of-fit tests revealed significant differences (*p* = 0.000) between the responses for all items (11–18) in section 2 of the survey (see Table 3).

### 4.2. Attitudes of UK Residents on the Import and Sale of Fur in the UK

#### 4.2.1. Knowledge about the Sale of Fur Products in the UK

Table 4 illustrates the respondents’ knowledge about the sale of fur products in the UK. The majority (59.6%) of respondents are aware that farming animals for their fur is banned in the UK. A larger majority (80.5%) are aware that fur from farmed species is imported and sold in the UK. Two-thirds (66.7%) of respondents are aware that fur from wild-caught species is imported and sold in the UK. A small minority (7.8%) of respondents have knowingly purchased products made from real fur in the past five years. Of those respondents (*n* = 22), when buying the product, 36.4% (*n* = 8) considered the country of origin of the fur product, 68.2% (*n* = 15) considered the species and 50% (*n* = 11) considered whether the animal was farmed or wild-caught. Chi-squared goodness-of-fit tests revealed significant differences (*p* < 0.01) between the responses for all items (19–22) presented below in Table 4.

Item 24 was an open-ended question: ‘If you have purchased a product made from real fur, please state your reason for doing so’. Participants responded with a range of answers including presuming it was faux fur, purchasing vintage items, purchasing rabbit fur for cat toys (and believing rabbit fur is a by-product of the meat industry), not having ethical concerns about whether the fur came from well-regulated farms, purchasing a Canada Goose jacket before realising the cruelty involved, the perceived better quality than synthetic alternatives and because a jacket simply looked appealing and fashionable.

#### 4.2.2. Attitudes to the Sale of Fur in the UK

Table 5 summarises the responses about attitudes to the sale of fur products in the UK. Almost all respondents (95.8%) believe that all fur products should be labelled as ‘real’ or ‘synthetic’. A small majority of respondents (4.9%) approve of retailers that sell fur, with 14.8% neutral and 80% disapproving. Related to this, around three-quarters (77.4%) disagree that buying real fur is morally acceptable, with 7.4% agreeing and 14.8% remaining neutral.

Table 5 includes two items that compare fur farming to farming for the production of meat and compare wearing fur to wearing leather. Question 29 states ‘There is no moral difference between farming animals such as pigs and chickens for meat and farming animals such as mink and foxes for fur’. In response, around one-third (35.9%) agreed, around half (52.5%) disagreed and 11.3% were neutral. Question 30 states ‘There is no moral difference between wearing real fur and wearing leather’. In response, around one-third (34.9%) agreed, just under half (45.8%) disagreed and 19% were neutral.

Farming animals for fur products is banned across the UK. However, the UK continues to permit the import and sale of fur products. A substantial majority (83.4%) disagreed with this position, while 6% agreed and 10.2% were neutral. The fur industry has implemented welfare schemes, such as WelFur, and the item ‘I oppose the use of real fur regardless of the welfare schemes implemented by the fur trade’ explored this issue. In response, three-quarters (75%) of respondents agreed with the statement, 14.8% disagreed and 9.9% were neutral. These findings could relate either to the species being used for fur or to the purpose of farming. Question 33 explored this issue with the statement ‘I oppose the use of real fur regardless of the species used’. A substantial majority (77.8%) were opposed to the use of real fur regardless of the species used, with 13.4% not opposed and 8.5% neutral.

Two items in Table 5 explore the idea of wearing fur products for fashion. A substantial majority (83.4%) agreed that farming and killing animals for commercial use in fashion is wrong, with 6.3% disagreeing and 9.9% neutral. A larger majority (91.6%) agreed that trapping and killing wild animals for commercial use in fashion is wrong, with 2.9% disagreeing and 5.3% neutral. Government regulation is often used to legislate on and enforce welfare standards in various contexts of animal use. Item 36 explored this issue with the statement, ‘No regulatory changes to animal welfare standards could provide animals farmed for their fur with a ‘life worth living’’. Around three-quarters (74%) agreed with this statement, with 14.1% disagreeing and 11.6% neutral.

Chi-squared goodness-of-fit tests revealed significant differences (*p* = 0.000) between the responses to items 26–36 in section 3 of the survey (see Table 5).

### 4.3. Do UK Residents Support a Ban on the Import and Sale of Fur in the UK?

**Figure 1:**
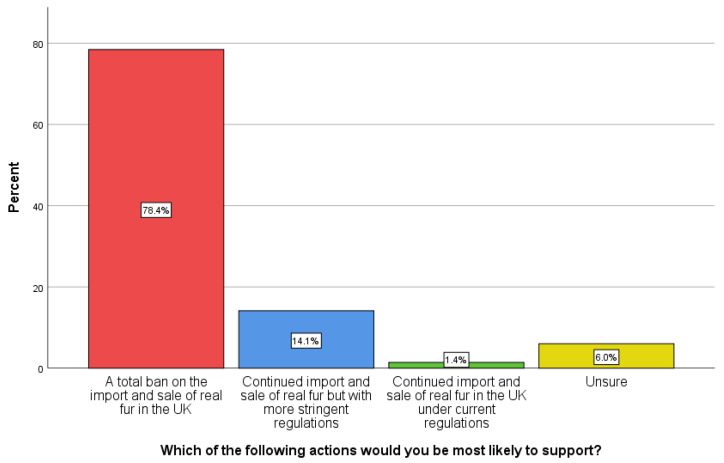
UK residents’ views on government regulation on the import and sale of fur products.

Respondents were asked about government policy on the import and sale of fur products. Fur farming is banned across the UK but the sale and import of fur products are permitted. The UK left the EU in 2020 and the British government is exploring a potential ban on the import and sale of fur post-Brexit. Figure 1 shows that a substantial majority (78.4%) support a total ban on the importation and sale of real fur in the UK. Around one in seven respondents (14.1%) support the continued import and sale of real fur but with more stringent regulations. Around 6% (6.0%) are unsure and a very small minority (1.4%) support the status quo. A chi-squared goodness-of-fit test revealed there was no significant difference between the responses for male and female respondents (*p* > 0.094) (Chi statistic 6.4, df 3, N 282), although there were low response rates (<5) in some answer categories.

## 5. Discussion

### 5.1. Beliefs about the Welfare of Animals Killed for Their Fur

In this research, respondents generally concurred with the belief that animals farmed for their fur experience poor welfare. For instance, 72.3% disagreed that animals on fur farms have a life worth living. To not have a life worth living means that fur-farmed animals would be better off dead or having never been born [38]. Only a small minority of respondents, at 4.3%, were of the view fur-farmed animals have a good life. Furthermore, the majority of respondents, at 75.2%, agreed that fur farms cannot meet the welfare needs of species such as mink and foxes. These responses are consistent with expert scientific reviews. For instance, the EU SCAHAW [17] reported major problems in fur farming, while Picket and Harris [39] asserted that the species subject to fur farming are not domesticated and are thus ill-adapted to being kept in highly restricted environments.

The results also revealed substantial majorities of the respondents who concurred with the belief that both fur-farmed and wild-caught animals suffer during slaughter. For fur-farmed animals, 77.8% agreed they are not killed humanely. For wild-caught animals, 91.2% disagreed that animals caught in traps are killed humanely. Then, for leg-hold traps, 96.4% of respondents agreed they cause suffering to species such as the coyote and lynx.

### 5.2. Knowledge about the Sale of Fur Products in the UK

Fur farming has been banned in England and Wales under the Fur Farming (Prohibition) Act since 2000 and under parallel legislation in Scotland and Northern Ireland since 2002. Despite this, at 59.6%, only a small majority of respondents were aware that farming animals for their fur is banned in the UK. This figure may in part be explained by the continued import and sale of fur products in the UK, despite the national prohibition on farming animals for their fur. A far higher proportion, 80.5%, was aware that fur from farmed animals is imported and sold in the UK. This figure might be explained simply because respondents as consumers are aware that fur products are marketed in some UK stores. The high proportion might also relate to respondents being aware of high-profile campaigns against stores that sell fur. For instance, the ‘House of Horror’ campaign against the UK department store House of Fraser in 2019 was covered widely in national media [40,41].

Two-thirds (66.7%) of respondents were aware that fur from wild-caught species is imported and sold in the UK. This figure is lower than the 80.5% of respondents that were aware that fur from farmed animals is available to buy. The lower 66.7% proportion may have arisen as some respondents are aware of UK-wide bans on certain fur products and methods of trapping. With some exemptions, the EU has banned the import and sale of seal fur and also the import of fur products harvested using the steel-jaw leg-hold trap. Hence, some respondents possibly believed there were wider prohibitions on the import and sale of wild-caught fur products.

### 5.3. Attitudes toward Fur and Its Import and Sale within the UK

A large majority of respondents, 92.2%, had not knowingly purchased real fur products in the five years before completing the questionnaire. For those that did knowingly purchase fur products, around one-third (36.4%) considered the country of origin, around two-thirds (68.2%) considered the species and half (50%) considered whether the animal was farmed or wild-caught when purchasing the product. These figures suggest that a significant proportion of those that do purchase fur products nevertheless consider the ethics of the industry. China, as a major fur producer, has no national animal welfare law and poor animal welfare standards in general. The EU, another major fur producer, has at least some protection for fur-farmed animals. Council Directive 98/58/EC protects all animals kept for farming purposes, including those reared for fur. Council Regulation (EC) no. 1099/2009 on the protection of animals at the time of killing also applies to fur-farmed animals.

Participants’ responses considering the species and whether the fur product is farmed or wild-caught are more difficult to interpret. For the former, the consideration of species at the time of purchase may not relate to animal welfare but to considerations about fashion and aesthetics, as the questionnaire item did not stipulate welfare-related reasons. For the latter, responses to whether a fur product is farmed or wild-caught are also interesting to interpret. If respondents were considering the welfare of the animal, they may have considered that wild-caught fur is more ethical because at least the animal was free to live naturally until the point in time when it was trapped. Alternatively, respondents might have considered that morally it is preferable to purchase a fur product from an animal that was bred and raised specifically for fur production. A further explanation is that some participants may perceive fur from wild-caught animals to be in some sense more authentic than that produced from farmed animals. These factors influencing the purchasing choices of those that consume fur products are perhaps an area for further research.

A final point to make is that responses to later parts of the survey may have been influenced by question 19, ‘Are you aware that farming animals for their fur is banned in the UK?’. This item was included before further questions assessing attitudes to the fur trade. Question 19 could be considered an information treatment that may have negatively influenced participants’ responses, especially those individuals who were not aware of the UK ban on fur farming.

### 5.4. Labelling and the Farming of Fur vs. Food and Fur vs. Leather

Wearing fur has been highly controversial in the UK and much of the western world since high profile campaigns in the 1970s and 1980s onwards. Consider, for instance, the iconic ‘I’d rather go naked than wear fur’ campaign by the People for the Ethical Treatment of Animals (PETA) [42]. Welfare issues associated with fur farming have also been brought to light in several high-profile investigations in recent years, including an exposé of Finland’s so-called ‘monster foxes’ [43]. Given that the fur industry has become so controversial, it is perhaps not surprising that an overwhelming majority of respondents, 95.9%, believed that all fur products should be labelled as real or synthetic. The figure is far higher, for example, than the 80% of respondents who did not approve of fur retailers or the 77.4% who believed that buying real fur is morally unacceptable. The figures suggest that many respondents who approve of consuming fur for fashion believe, at the same time, that the product should be properly labelled as real fur.

The questionnaire included two items that offer insight into how people view the ethics of purchasing and wearing fur in comparison with consuming meat and dairy products or wearing leather items. Just over half of respondents, at 52.5%, believed there to be a moral difference between farming pigs and chickens for meat and farming mink and foxes for fur. In the UK, the overwhelming majority of society consumes meat and other animal products, while only 3% identify as vegetarian and 1% vegan [44]. Furthermore, many believe that consuming meat and other animal products is necessary for their health [45]. For these reasons, many of the 52.5% that believe there is a moral difference between farming pigs and chickens for meat and farming mink and foxes for fur may hold this belief based on considerations related to necessity. That is to say, they may believe that farming animals for food production is necessary, but in contrast, farming animals for fur production is unnecessary. Thus, the moral difference likely relates to the purpose of the product. Indeed, fur farming was prohibited in the UK in a large part because it was perceived by Parliament to cause unnecessary suffering [46]. Fur as a commodity is unnecessary for British consumers and there is good scientific evidence that the production of it causes suffering [17].

Taking a different view, the respondents’ belief in a moral difference between farming pigs and chickens and farming mink and foxes may have related not to the purpose but the species. Scientific reports and journal articles have suggested that fur farming is inherently problematic. Pickett and Harris [39], for instance, assert that mink and foxes are not domesticated and remain essentially wild. The authors propose they have been artificially selected for the quality of their fur but not for docility or similar traits, as pigs, chickens and other farmed species have for millennia. This leads to major welfare problems in mink, foxes and other commonly used species in fur farming. Campaigns by groups such as HSI UK [16] and the Fur Free Alliance [47] have promoted public knowledge of the claim that fur farming is inherently cruel, not only due to the nature of the species involved but also because the conditions that animals are kept in on fur farms would be unacceptable for any animal, whether domesticated or not, and whether farmed for food or fur. Thus, participants may have been aware of this message from campaigning organisations when they were completing the questionnaire.

The survey statement, ‘There is no moral difference between wearing fur and wearing leather’, produced mixed responses in the questionnaire results. Just over one-third (35.9%) agreed, around half (52.5%) disagreed and 11.3% were neutral. In effect, substantially fewer respondents object morally to the leather industry compared to the fur industry. In the UK, while only a small minority of the population wears fur products, the majority wear leather. Furthermore, while fur is generally considered a luxury item, wearing leather shoes, for instance, is for a large proportion of British society an everyday habit. The production of leather and fur differs in two key respects. First, most leather worn in the UK is produced from cattle (*Bos Taurus*). Cattle have been farmed for millennia, and unlike fur-farmed species, are domesticated animals [48]. Secondly, a significant proportion of leather is produced as a by-product of the beef and dairy industries, though there are still major ethical concerns about the leather industry [49]. Hence, the 52.5% of the sample who responded that there is a moral difference between wearing fur and leather may have done so considering the species/domestication and/or due to beliefs about leather being a by-product of food farming. In contrast, the 35.9% that responded to say there is no moral difference may have done so considering the sentience shared by cows and fur-farmed species and/or based on their awareness of alternative materials to leather that are suitable for footwear and other clothing traditionally made from leather.

### 5.5. Stated Preference and Revealed Preference in the Public Consumption of Fur Products

We stated earlier in the paper that HSI UK has reported that the UK imports the equivalent of two million animals each year. Surveys conducted between 1997 and 2021, as reviewed in Section 2 of this paper, found that the public consistently opposed the fur industry. In a 2020 HSI UK-commissioned YouGov poll (*n* = 1682), 72% of the British public supported a ban on the import and sale of fur, with only 16% opposed to a ban [33]. In the same poll, 93% of respondents had either never worn fur or no longer wore fur, with only 3% stating they currently wore fur.

Despite such figures and the ban on fur farming across the UK, HSI UK has estimated that the UK continues to import the equivalent of two million whole furs each year [16]. The surveys reviewed in Section 2 of this paper, as well as our questionnaire investigating people’s beliefs and attitudes about the welfare of fur-farmed species and the import and sale of fur products in the UK, all report participants’ stated preferences. However, these stated preferences may overestimate their ‘true’ preferences when actual purchasing behaviour is considered, i.e., the participants’ revealed preferences. Academic studies have demonstrated a discrepancy between buying and voting behaviour in the context of animal welfare [50,51]. 

The reason for the potential gap between revealed and stated preferences relates to social desirability bias. For sensitive issues such as animal welfare, research participants may overstate their opposition to certain practices, such as those within the fur industry, at least when compared to their actual purchasing behaviour. In an approach that holds potential, indirect survey methods can be used to somewhat overcome such social desirability bias [52,53]. Investigating the impact of social desirability bias on attitudes to the fur industry may represent an area for future research.

### 5.6. Prohibiting the Import and Sale of Fur in the UK Post-Brexit

Fur farming has been banned in England and Wales under the Fur Farming (Prohibition) Act since 2000 and under parallel legislation in Scotland and Northern Ireland from 2002. Despite this, the UK continues to import fur products equivalent to two million animals per year [16]. At the time of writing in January 2022, the UK government is exploring a potential ban on the import and sale of fur post-Brexit. In this survey, a large majority of respondents (83.4%) believed it was not morally acceptable for the UK Government to ban fur farming in Britain and yet continue to import and sell fur from producers overseas. The respondents were asked to select one of four options on the UK policy for the import and sale of fur products. A substantial majority, at 78.5%, supported a total ban on the import and sale of fur in the UK. The next most frequent response, at 14.1%, was the continued import and sale of fur but with more stringent regulations. Only 1.4% of respondents favoured continuing the status quo for policy on the fur trade, with a further 6% unsure.

Arguably, the status quo concerning the governmental fur policy in the UK is contradictory. The UK ban was based on scientific research, public opinion against fur and the problematic ethics of causing suffering to sentient animals for fashion. Despite this, the UK continues to import an estimated equivalent of around two million whole furs each year, which represents a glaring contradiction. In support of overcoming that by extending the ban to imports and sales, this research has corroborated earlier research on UK public attitudes that are against fur. Polls have found that a significant majority of British citizens are opposed to fur farming and support a full ban on the import and sale of fur products in the UK [32,33]. The findings of this research further revealed that a large majority, 78.5%, support a full ban.

## 6. Conclusions

Fur has been a highly controversial issue in the UK and much of the western world since the second half of the 20^th^ century. Globally, 85% of fur is produced on farms from species such as mink and foxes and 15% is harvested from wild-caught animals such as coyotes and raccoons. China, the US, Canada and the EU are major fur producers. Scientific reviews have found major welfare issues in species such as mink and foxes farmed for their fur. The harvesting of fur from wild animals such as coyotes—for instance, using leg-hold traps—also causes severe suffering. The potential for devastation to public health and human society, on a global scale, has placed a further spotlight on fur farming. Mink and other fur-farmed species can become infected and transmit SARs-CoV-2, the virus that causes COVID-19, to humans. Since 2020, there have been outbreaks on 400 mink farms in Europe and America. Over 20 million fur-farmed animals have been culled on European farms, and EU countries such as the Netherlands have brought forward bans on fur farming.

Fur farming was banned in England and Wales under the Fur Farming (Prohibition) Act in 2000 and under parallel legislation in Scotland and Northern Ireland in 2002. Despite this, as a member of the EU, the UK continued to import and sell fur products. It has been estimated that the UK imported the equivalent of at least two million whole furs in 2018. The UK Government has stated that it was unable to ban the import and sale of fur as a member of the EU under the ‘free movement of goods’ principle. The UK left the EU in January 2020, and Brexit, therefore, presents an opportunity for the UK to ban the import and sale of fur products. Looking ahead, the British government is exploring a potential ban on the import and sale of fur now it is making changes post-Brexit.

This paper reviewed UK surveys on public attitudes to fur from 1997–2021. The surveys revealed a consistent and growing majority opposed to the fur industry. For instance, an RSPCA-commissioned poll in 1997 revealed that 73% of people disapproved of fur. Ten years later, a 2007 RSPCA poll found 93% of British adults would refuse to wear fur. Then, almost a decade on, a 2016 HSI UK poll found that only 12% of respondents felt it acceptable to buy and sell fur from foxes and mink. Furthermore, a 2020 HSI UK poll found that 72% of the British public would support a ban on the import and sale of fur in the UK, and 93% of those polled had either never worn fur or no longer wore it.

This research used an online questionnaire (*n* = 326) to investigate in greater depth the beliefs of UK residents about the welfare of fur-farmed and wild-caught animals and people’s attitudes toward the fur trade. Respondents had a high level of awareness of welfare issues concerning the fur trade. For instance, 75.2% agreed that fur farms cannot meet the welfare needs of species such as mink and foxes. Furthermore, 96.4% of respondents agreed that leg-hold traps cause suffering to species such as the coyote and lynx. Only 59.6% of respondents were aware that the UK has banned the farming of animals for fur. A far higher proportion, 80.5%, was aware that fur from farmed animals is imported and sold in the UK. A considerable 95.9% of the sample responded to say that all fur products should be labelled as real or synthetic, given the controversial nature of the fur trade. A large majority of respondents, at 83.4%, agreed it is not morally acceptable for the UK Government to ban fur farming and yet continue to import and sell fur from producers overseas. A substantial majority, at 78.5%, supported a total ban on the import and sale of fur in the UK.

## Figures and Tables

**Table 1:** A summary of survey results on UK public attitudes to fur from 1997 to 2021.

Year	Source	Results
1997	Ipsos MORI poll (commissioned by Marie Claire and the RSPCA) [27]	Survey of 1946 adults (aged 15+ in 173 sampling points throughout Great Britain in June 1997. Interviews were conducted face-to-face in-home): 87% of adults would never wear real fur. Of these, 73% thought it was wrong or disapproved, 12% said it was too expensive, 8% cited it as unfashionable and 6% thought other people would disapprove.
2007	RSPCA poll [28]	93% of British adults refused to wear real fur; 43% checked labels to see if the fur was real or fake; 92% believed fur should be labelled as real or fake; 91% would not buy fur even if it was cheap; 61% thought celebrities should not wear real fur; 61% thought there was a moral difference between animals farmed for meat and animals raised for fur.
2010	TNS PhoneBus poll (commissioned by RSPCA) [29]	A TNS opinion poll was conducted in Great Britain via TNS PhoneBus, a telephone Omnibus survey. A representative sample of 2004 adults aged 16 and above were interviewed in September and October 2010. 95% refused to wear real fur and 93% thought products should be clearly labelled as real or fake fur.
2014	YouGov poll (commissioned by FOUR PAWS) [30]	Survey of 2081 individuals (aged ≥18). Fieldwork was conducted online in January 2014. The figures were weighted to be representative of all UK adults (aged 18+). 62% of the UK population preferred to buy from retailers that did not sell animal fur products. 74% of the interviewed individuals thought that using animals to produce fur for the fashion industry was wrong.
2016	YouGov poll (commissioned by HSI UK) [31]	Survey of 2051 individuals (aged ≥18). <10% felt it was acceptable to be able to buy and sell products containing domestic dog fur (7%), seal fur (8%) or cat fur (9%). Between 8% and 12% felt it was acceptable to be able to buy and sell products containing fur from: foxes (12%), mink (12%), chinchillas (9%), raccoon dogs (8%) and coyotes (8%). 20% felt it was acceptable to be able to buy and sell products containing rabbit fur.
2018	YouGov poll (commissioned by HSI UK) [32]	“To what extent, if at all, would you support or oppose a ban on the import and sale of all animal fur in the UK?” Survey of 1594 people, conducted online in February 2018. The figures were weighted to be representative of all GB adults (aged 18+). 69% of the British public strongly supported (46%) or tended to support (23%) a ban. 8% opposed a ban, composed of 3% who strongly opposed it and 5% who tended to oppose it. 18% stated they would neither support nor oppose a ban, and the remaining 6% did not know.
2020	YouGov poll (commissioned by HSI UK) [33]	“To what extent, if at all, would you support or oppose a ban on the import and sale of animal fur in the UK?” Survey of 1682 people, conducted online in 2020. The figures were weighted to be representative of all GB adults (aged 18+). 72% of the British public strongly supported (52%) or tended to support (21%) a ban. 16% were opposed, composed of 8% who strongly opposed it and 8% who tended to oppose it. 12% did not know. “Do you wear, or have you ever worn, real animal fur?” A total of 93% of those polled either had never worn fur or no longer wore it (10% had worn real fur in the past but no longer did so, while 83% did not wear fur and never had); 3% currently wore real fur; 5% did not know.
2021	YouGov poll (commissioned by HSI UK) [34]	Survey of 1647 people, conducted in 2021. 93% considered it unacceptable to keep foxes for their whole lives in wire cages measuring 1–1.5 m^2^. 92% considered it unacceptable to trap wild animals (e.g., coyotes) in leg-hold traps. 92% considered it unacceptable to kill foxes by anal/vaginal electrocution.
2021	YONDER poll (commissioned by HSI UK) [35]	“To what extent, if at all, would you support or oppose a ban on the import and sale of animal fur in the UK?” Survey of 2026 people, conducted in 2021. 72% of the British public strongly supported (52%) or tended to support (20%) a ban. 12% were opposed, composed of 7% who strongly opposed it and 4% who tended to oppose it.

**Table 2:** Structure of the survey including items and response options in sections 2 and 3.

Section	Questions’ Wording	Response Type/Measurement Scale
Section 2—Beliefs about the welfare of animals killed for their fur	I believe animals on fur farms generally have a life worth living	Likert scale: strongly agree, agree, neutral, disagree, strongly disagree
	I believe animals on fur farms generally have a good life	
	I believe animals farmed for their fur are killed humanely	
	I believe animal welfare standards on fur farms are well-regulated	
	I believe fur farms can meet the welfare needs of species such as the mink and fox	
	I believe cages provide fur farm species such as the mink and fox with a good level of welfare	
	I believe leg-hold traps used to catch wild species such as the coyote and lynx do not cause suffering	
	I believe animals caught in traps in the wild are killed humanely	
Section 3—Attitudes toward buying fur and the import and sale of fur in the UK	Are you aware that farming animals for their fur is banned in the UK?	Yes/no
	Are you aware that fur from farmed species is imported and sold in the UK?	
	Are you aware that fur from wild-caught species is imported and sold in the UK?	
	In the past five years, have you knowingly purchased any products made from real fur?	
	If you purchased a product made of fur, did you consider any of the following? (Country of origin of the product; Species of animal used to make the product; Whether the animal was farmed or wild-caught; N/A—I did not purchase a real fur product)	Multiple choice, four response options
	If you have purchased fur, please state your reasons for doing so?	Open-text response
	If you were to purchase a fur product, which of the following would be the most important to you? (Country of origin of the product; Species of animal used to make the product; Whether the animal had received a good level of welfare; Whether the animal was killed humanely; Whether the animal was farmed or wild-caught; N/A—I would not purchase a fur product)	Multiple choice, six response options
	I believe all fur products should be labelled as ‘real’ or ‘synthetic’	Likert scale: strongly agree, agree, neutral, disagree, strongly disagree
	I approve of retailers that sell real fur	
	Buying real fur is morally acceptable	
	There is no moral difference between farming animals such as pigs and chickens for meat and farming animals such as mink and foxes for fur	
	There is no moral difference between wearing real fur and wearing leather	
	It is morally acceptable for the UK government to ban fur farming and continue to import and sell fur from producers overseas	
	I oppose the use of real fur regardless of the welfare schemes implemented by the fur trade	
	I oppose the use of real fur regardless of the species used	
	Farming and killing animals for commercial use in fashion is wrong	
	Trapping and killing wild animals for commercial use in fashion is wrong	
	No regulatory changes to animal welfare standards could provide animals farmed for their fur with ‘a life worth living’	
	Which of the following actions would you be most likely to support? (A total ban on the importation and sale of real fur in the UK; Continued importation and sale of real fur but with more stringent regulations; Continued importation and sale of real fur in the UK under current regulations; Unsure).	Multiple choice, four response options

**Table 3:** Beliefs of UK residents about the welfare of fur-farmed and wild-trapped animals. Bold indicates aggregated agreement or disagreement responses. Significance column = *p*-value (Chi-squared statistic, df, N).

Q	Statement	Strongly Agree	Agree	Neutral	Disagree	Strongly Disagree	Chi-Squared
11	I believe animals on fur farms generally have a life worth living	12.7% (39)	8.1% (25)	6.8% (21)	17.9% (55)	54.4% (167)	0.000 (241.1, 4, 305)
		20.8% (64)			72.3% (222)		
12	I believe animals on fur farms generally have a good life	1% (3)	3.3% (10)	7.2% (22)	22.5% (69)	66.1% (203)	0.000 (456.0, 4, 305)
		4.3% (13)			88.6% (272)		
13	I believe animals farmed for their fur are killed humanely	2.3% (7)	7.2% (22)	12.7% (39)	25.7% (79)	52.1% (160)	0.000 (247.9, 4, 305)
		9.5% (29)			77.8% (239)		
14	I believe animal welfare standards on fur farms are well-regulated	1% (3)	5.2% (16)	18.6% (57)	25.4% (78)	49.8% (153)	0.000 (233.5, 4, 305)
		6.2% (19)			75.2% (231)		
15	I believe fur farms can meet the welfare needs of species such as the mink and fox	1.6% (5)	4.9% (15)	11.4% (35)	27.7% (85)	54.4% (167)	0.000 (291.9, 4, 305)
		6.5% (20)			82.1% (252)		
16	I believe cages provide fur farm species such as the mink and fox with a good level of welfare	0.3% (1)	1.6% (5)	7.2% (22)	21.2% (65)	69.7% (214)	0.000 (520.6, 4, 305)
		1.9% (6)			90.9% (279)		
17	I believe leg-hold traps used to catch wild species such as the coyote and lynx do not cause suffering	0.3% (1)	1% (3)	2.3% (7)	14% (43)	82.4% (254)	0.000 (558.4, 3, 305)
		1.3% (4)			96.4% (297)		
18	I believe animals caught in traps in the wild are killed humanely	1.3% (4)	2.3% (7)	5.2% (16)	17.9% (55)	73.3% (225)	0.000 (577.9, 4, 305)
		3.6% (11)			91.2% (280)		

**Table 4:** Knowledge about government regulation and the fur trade in the UK. Significance column = *p*-value (Chi-squared statistic, df, N).

Question	Yes	No	Chi-Squared
Are you aware that farming animals for their fur is banned in the UK?	59.6% (168)	40.4% (114)	0.001 (10.7, 1, 283)
Are you aware that fur from farmed species is imported and sold in the UK?	80.5% (227)	19.5% (55)	0.000 (105.8, 1, 283)
Are you aware that fur from wild-caught species is imported and sold in the UK?	66.7% (188)	33.3% (94)	0.000 (31.9, 1, 283)
In the past five years, have you knowingly purchased any products made from real animal fur? (This includes accessories or garments with fur trimmings.)	7.8% (22)	92.2% (260)	0.000 (201.8, 1, 283)

**Table 5:** Attitudes of UK residents toward the fur trade. Bold indicates aggregated agreement or disagreement responses. Significance column = *p*-value (Chi-squared statistic, df, N).

Question	Strongly Agree	Agree	Neutral	Disagree	Strongly Disagree	Chi-Squared
26. I believe all fur products should be labelled as ‘real’ or ‘synthetic’	77.8% (221)	18% (51)	1.8% (5)	0.7% (2)	1.4% (4)	0.000 (626.7, 4, 283)
	95.8% (272)			2.1% (6)		
27. I approve of retailers that sell real fur	2.1% (6)	2.8% (8)	14.8% (42)	17.3% (49)	62.7% (178)	0.000 (352.1, 4, 283)
	4.9% (14)			80% (227)		
28. Buying real fur is morally acceptable	2.1% (6)	5.3% (15)	14.8% (42)	15.1% (43)	62.3% (177)	0.000 (339.0, 4, 283)
	7.4% (21)			77.4% (220)		
29. There is no moral difference between farming animals such as pigs and chickens for meat and farming animals such as mink and foxes for fur	16.5% (47)	19.4% (55)	11.3% (32)	26.1% (74)	26.4% (75)	0.000 (23.7, 4, 283)
	35.9% (102)			52.5% (149)		
30. There is no moral difference between wearing real fur and wearing leather	17.6% (50)	17.3% (49)	19% (54)	31% (88)	14.8% (42)	0.000 (23.1, 4, 283)
	34.9% (99)			45.8% (130)		
31. It is morally acceptable for the UK government to ban fur farming and continue to import and sell fur from producers overseas	2.1% (6)	3.9% (11)	10.2% (29)	30.6% (87)	52.8% (150)	0.000 (265.9, 4, 283)
	6% (17)			83.4% (237)		
32. I oppose the use of real fur regardless of the welfare schemes implemented by the fur trade	54.9% (156)	20.1% (57)	9.9% (28)	9.5% (27)	5.3% (15)	0.000 (235.1, 4, 283)
	75% (213)			14.8% (42)		
33. I oppose the use of real fur regardless of the species used	58.1% (165)	19.7% (56)	8.5% (24)	8.8% (25)	4.6% (13)	0.000 (277.6, 4, 283)
	77.8% (221)			13.4% (38)		
34. Farming and killing animals for commercial use in fashion is wrong	66.5% (189)	16.9% (48)	9.9% (28)	4.9% (14)	1.4% (4)	0.000 (406.4, 4, 283)
	83.4% (237)			6.3% (18)		
35. Trapping and killing wild animals for commercial use in fashion is wrong	75.4% (214)	16.2% (46)	5.3% (15)	1.8% (5)	1.1% (3)	0.000 (568.1, 4, 283)
	91.6% (260)			2.9% (8)		
36. No regulatory changes to animal welfare standards could provide animals farmed for their fur with ‘a life worth living’	51.8% (147)	22.2% (63)	11.6% (33)	9.9% (28)	4.2% (12)	0.000 (204.5, 4, 283)
	74% (210)			14.1% (40)		

## Data Availability

The data presented in this study are openly available in FigShare at https://eur02.safelinks.protection.outlook.com/?url=https%3A%2F%2Fdoi.org%2F10.6084%2Fm9.figshare.19195769.v1&data=04%7C01%7CSteven.McCulloch%40winchester.ac.uk%7C93116d7ce5f949db511c08d9f2d60bbe%7C9ef0ad7deaab48a5a07afbb82033fa03%7C0%7C0%7C637807823388540490%7CUnknown%7CTWFpbGZsb3d8eyJWIjoiMC4wLjAwMDAiLCJQIjoiV2luMzIiLCJBTiI6Ik1haWwiLCJXVCI6Mn0%3D%7C3000&sdata=JA79kDwPIv9IDm7JcXaRU%2FtPEw28X9x9JjWh8R9dF7Q%3D&reserved=0, accessed on 10 November 2021. Citation: Halliday, C.; McCulloch, S.P. Excel Data from SurveyMonkey CH,SM.xlsx 2022.

## Supplementary Materials

The following supporting information can be downloaded at: https://www.mdpi.com/article/10.3390/ani12050538/s1, File S1: Survey.
