# Supplementary material for: Beliefs and Attitudes of British Residents about the Welfare of Fur-Farmed Species and the Import and Sale of Fur Products in the UK
Source: Animals (Basel). 2022 Feb 22;12(5):538. doi: 10.3390/ani12050538 (PMC8908824; doi:10.3390/ani12050538)
Supplement: Supplementary file 1 [file animals-12-00538-s001.zip › animals-1510792-supplementary.pdf]

animals

MDPI

Supplementary File S1: Survey

Survey section one – demographic information

Please check the box below to indicate you have read and understood the above information and consent to continue with the survey. (tick box)

What is your gender?
Female
Male
Prefer not to say
Other (please specify)

Copyright: © 2022 by the authors. Licensee MDPI, Basel, Switzerland. This article is an open access article distributed under the terms and conditions of the Creative Commons Attribution (CC BY) license (https://creativecommons.org/licenses/by/4.0/).

What is your age?
16-19
20-29
30-39
40-49
50-59
60-69
70-79
80+
Prefer not to say

What is your nationality?
Northern Irish
Scottish
English
Welsh
Other (please specify)

Which region of the UK do you live in?
Northern Ireland
Scotland
Wales
East of England
East Midlands
South East
South West
North East
North West

West Midlands
Yorkshire and the Humber
Other (please specify)

**Which is your ethnic group?**
White
Asian/Asian British
Mixed/multiple ethnic groups
Black/African/Caribbean/Black British
Chinese
Arab
Prefer not to say
Other (please specify)

**Which is your religion?**
No religion
Christian (including Church of England, Catholic, Protestant and all other Christian denominations)
Buddhist
Hindu
Jewish
Muslim
Sikh
Prefer not to say

**Which is the highest level of education you have completed?**
Primary school
Secondary school (GCSE or equivalent)
College (A Level or equivalent)
University (Bachelors degree or equivalent)
University (Masters degree or equivalent)
University (PhD or equivalent)
Prefer not to say
Other (please specify)

**Which is your current employment status?**
Full-time employment
Part-time employment
Self-employed
Unemployed
Student
Retired
Prefer not to say

**Which is your current annual income (before tax)?**

Less than £15,000
£15,001 – £20,000
£20,001 – £30,000
£30,001 – £40,000
£40,001 – £50,000
£60,001 – £70,000
£70,001 – £100,000
£101,000 +
Prefer not to say
Other (please specify)

Survey section 2: beliefs about the welfare of animals killed for their fur

This section of the survey focuses on your beliefs about the welfare of animals on fur farms and those trapped and killed in the wild for their fur.

Please note: Animals in relation to fur farming refers to species such as mink, fox, raccoon dog, rabbit, and chinchilla. Animals in relation to those trapped in the wild refer to species such as coyote, lynx, beaver and raccoon.

An animal's quality of life can be classified as: 'a life not worth living', a 'life worth living' and 'a good life'.

The concept of 'a good life' recognises the distinction that an animal's quality of life is over and beyond that of a life worth living.

Q 11 – 18

Question	Strongly Agree	Agree	Neutral	Disagree	Strongly Disagree
I believe animals on fur farms generally have a life worth living					
I believe animals on fur farms generally have a good life					
I believe animals farmed for their fur are killed humanely					
I believe animal welfare standards on fur farms are well regulated					
I believe fur farms can meet the welfare needs of species such as mink and fox					
I believe cages provide fur farm species such as mink and fox with a good level of welfare					
I believe leg-hold traps used to catch wild species such as coyote and					

lynx, do not cause suffering	
I believe animals caught in traps in the wild are killed humanely	

Survey section 3: attitudes towards buying fur and the importation and sale of fur in the UK

This section of the survey asks you about your attitudes towards the purchase of fur products and the sale and importation of fur products in the UK.

Please note: 'fur products' in the questions below refers to products made from the skin of animals which have been obtained either from fur farms or from animals trapped in the wild.

Q 19 – 22

Question	Yes	No
Are you aware that farming animals for their fur is banned in the UK?		
Are you aware that fur from farmed species is imported and sold in the UK?		
Are you aware that fur from wild caught species is imported and sold in the UK?		
In the past five years, have you knowingly purchased any products made from real animal fur? (This includes accessories or garments with fur trimmings.)		

Q23

If you purchased a product made from real fur, did you consider any of the following? (Multiple choice)	
Country of origin of the product	
Species of animal used to make the product	
Whether the animal was farmed or wild caught	
N/A – I did not purchase a real fur product	

Q24

If you have purchased a product made from real fur, please state your main reasons for doing so. (If you haven't purchased a fur product please enter 'N/A') (open text response)

Q25

If you were to purchase a product made from real fur, which of the following would be most important to you? (Multiple choice)	

Country of origin of the product
Species of animal used to make the product
Whether the animal had received a good level of welfare
Whether the animal was killed humanely
Whether the animal was farmed or wild caught
N/A – I would not purchase a fur product

Q26-36

Question	Strongly Agree	Agree	Neutral	Disagree	Strongly Disagree
I believe all fur products should be labelled as 'real' or 'synthetic'					
I approve of retailers that sell real fur					
Buying real fur is morally acceptable					
There is no moral difference between farming animals such as pigs and chickens for meat and farming animals such as mink and fox for fur					
There is no moral difference between wearing real fur and wearing leather					
It is morally acceptable for the UK government to ban fur farming and continue to import and sell fur from producers overseas					
I oppose the use of real fur regardless of the welfare schemes implemented by the fur trade					
I oppose the use of real fur regardless of the species used					
Farming and killing animals for commercial use in fashion is wrong					
Trapping and killing wild animals for commercial use in fashion is wrong					
No regulatory changes to animal welfare standards could provide animals farmed for their fur with 'a life worth living'					

Q37

**Which of the following actions would you be most likely to support? (Multiple choice)**
A total ban on the importation and sale of real fur in the UK
Continued importation and sale of real fur but with more stringent regulations
Continued importation and sale of real fur in the UK under current regulations
Unsure

End of survey.

Thank you for taking the time to complete this survey.
